# Minocycline in Treating Glioblastoma Multiforme: Far beyond a Conventional Antibiotic

**DOI:** 10.1155/2020/8659802

**Published:** 2020-09-17

**Authors:** Amir R. Afshari, Hamid Mollazadeh, Amirhossein Sahebkar

**Affiliations:** ^1^Department of Physiology and Pharmacology, Faculty of Medicine, North Khorasan University of Medical Sciences, Bojnurd, Iran; ^2^Natural Products and Medicinal Plants Research Center, North Khorasan University of Medical Sciences, Bojnurd, Iran; ^3^Halal Research Center of IRI, FDA, Tehran, Iran; ^4^Biotechnology Research Center, Pharmaceutical Technology Institute, Mashhad University of Medical Sciences, Mashhad, Iran; ^5^Neurogenic Inflammation Research Center, Mashhad University of Medical Sciences, Mashhad, Iran; ^6^School of Pharmacy, Mashhad University of Medical Sciences, Mashhad, Iran

## Abstract

One of the most lethal forms of CNS pathologies is glioblastoma multiforme (GBM) that represents high invasiveness, uncontrolled proliferation, and angiogenic features. Its invasiveness is responsible for the high recurrence even after maximal surgical interventions. Minocycline is a semisynthetic analog of tetracyclines with potential anti-inflammatory and anticancer effects, distinct from its antimicrobial activity. In this review, we highlight the importance and the cytotoxic mechanisms of minocycline on GBM pathophysiology. Considering the role of certain enzymes in autophagy, apoptosis, tumor cell invasion, and metastatic ability, the possible use of tetracyclines for cancer therapy should be investigated, especially GBM. The present study is, therefore, going to cover the main topics in minocycline pharmacology to date, encouraging its consideration as a new treatment approach for cancer and GBM.

## 1. Introduction

Tetracyclines, as broad-spectrum bacteriostatic antibiotics, work against pathologic microorganisms like rickettsia, chlamydia, and mycoplasma pneumonia, as well as against a wide range of Gram-positive, Gram-negative, and anaerobic bacteria [1]. The antibiotic characteristics of tetracyclines were defined at the end of the 1940s; however, several studies concentrated more recently on their nonantibiotic properties [2]. The second-generation semisynthetic tetracycline analog used for over 30 years is minocycline (7-dimethylamino-6-desoxytertracycline) [3].

This highly lipophilic molecule can easily cross the blood-brain barrier (BBB) and has demonstrated neuroprotective effects in experimental models of stroke, trauma, and neurodegenerative disorders like multiple sclerosis, Huntington disease, and Parkinson [4]. It is therefore not unexpected that a series of minocycline clinical trials for many central nervous system (CNS) disorders are now under progress [5]. While many pieces of evidence suggest a convergent activity of minocycline in the CNS inflammation and apoptosis inhibition via the suppression of microglial activation and inhibiting neural cell death [6, 7], the precise molecular targets of minocycline in cancer still have to be determined.

Mechanistically, minocycline seems to have potential biological activities including immunomodulatory, neuroprotective, antiapoptotic, and antiinflammatory effects [8–11] and inhibitory effects on proteolysis, angiogenesis, and metastasis of the tumor [12–14], independent of its antimicrobial activity. In many of these reports, the mechanisms for anti-inflammatory, immunomodulating, and neuroprotective effects of minocycline have been discussed, such as inhibitory impacts on the activities of inducible nitric oxide (NO) synthase (iNOS) and matrix metalloproteinases (MMPs), cyclooxygenase (COX)/lipoxygenase (LOX) inhibition, suppression of caspase-1/-3/-8 activation, inhibition of p38 mitogen-activated protein kinase (MAPK) phosphorylation, the blockage of poly (ADP-ribose) polymerase 1 (PARP-1) activation, downregulation of proapoptotic proteins (Bax, Bak, and Bid), an upregulation in antiapoptotic protein Bcl-2, consequently protecting the cells from apoptosis [15–17]. Nonetheless, these antiapoptotic effects come from disorders involving excessive apoptosis [18]. Cellular apoptosis is, however, strongly downregulated in cancer [19].

The most aggressive infiltrative glioma is glioblastoma multiforme (GBM), a group of primary tumors occurring from the CNS (nearly 70% of all tumors) [20, 21]. Typically, a three-way method is needed for the clinical treatment of GBM, including maximal surgical resection, adjuvant radiotherapy with concomitant, and maintenance chemotherapy (temozolomide, TMZ) [22]. Despite the widespread efforts to establish new therapies [23–25], GBM seems to have a high failure rate in the affected patients. Herein, throughout the present review, we endeavored to describe the anticancer impacts of minocycline in different preclinical and clinical models and further promote its evaluation as a potential therapeutic strategy in GBM, as an immune-response deregulated disease.

## 2. The Role of Minocycline in Cancer

The sequence of events that contribute to tumor cell invasion and metastasis is of significant importance for the prognosis of cancer patients [26]. Matrix metalloproteinases (MMPs) have been identified as the main proteolytic enzymes used to regulate the degradation of the extracellular matrix (ECM) [27]. It has been shown that tetracyclines and chemically modified tetracyclines, especially minocycline, suppress the activity of several MMPs [28], to induce cell growth suppression in numerous cancer cells. Masumori et al. have shown that minocycline could inhibit invasion and pulmonary metastasis, both *in vitro* and *in vivo* [29]. Interestingly, minocycline is extremely useful in reducing MMPs expression released by bone tumor cells [30]. In this regard, Niu et al. have suggested that combined use of celecoxib (a COX-2 inhibitor) and minocycline has promising inhibitory effects on the metastasis of breast cancer, as compared to celecoxib or minocycline alone [31].

MMPs also engage in controlling other nonmatrix targets in addition to their tissue-remodeling features, like cell-cell adhesion molecules, cell surface receptors, clotting factors, cell fusion, cytokines, and chemokines [32]. Interleukin-6 (IL-6) has been proven to be a significant nonmatrix target of MMPs in cancer growth and development by modulating many signaling molecules, in particular signal transducer and activator of transcription-3 (STAT-3) and extracellular signal-regulated kinase (ERK) 1/2. Therefore, as a therapeutic alternative for cancer, targeting IL-6 is progressively enticing. For instance, minocycline could suppress IL-6 as well as inhibiting invasion, migration, and adhesion capacity of ovarian cancer cells *in vitro* and *in vivo* [33]. Nuclear factor-kappa-B (NF-*κ*B), as a vital factor of inflammation, participated in the progression of cancer through cytokine production [33,34]. Weiler et al. have shown that minocycline by inhibiting the NF-*κ*B activation could reduce the tumor necrosis factor- (TNF-) *α*-induced cell fusion [35]. Notably, a hydroxyl-G6 PAMAM dendrimer-9-amino-minocycline conjugate (D-mino) increased the intracellular availability of the drug due to its rapid uptake, suppressed inflammatory cytokine such as TNF-*α* production, and reduced oxidative stress by suppressing NO production, which provides new options for targeted drug delivery to treat neurological disorders [36].

Also, minocycline exerts differential effects on the regulation of cytokine production by T cells and monocytes [37]. For instance, in ovarian cancer cells, minocycline orally administered is very useful in suppressing malignant ascites through targeting cytokines and growth factors necessary for the development and forming of a tumor [38]. Besides, minocycline attenuated lipopolysaccharide (LPS) stimulated degradation of inhibitor of kappa-B (I*κ*B)*α*, implying a possible anti-inflammatory role for NF-*κ*B transcriptional activity [39]. In another study of Ataie-Kachoie et al., it was found that minocycline targets the NF-*κ*B pathway through suppression of the transforming growth factor- (TGF-) *β*1-TAK1-I*κ*B kinase axis in ovarian cancer [40]. Furthermore, minocycline has beneficial effects on ovarian cancer by inhibition of p65 phosphorylation and nuclear translocation accompanied by downregulation of NF-*κ*B activity [41]. These findings have indicated the critical feature of the NF-*κ*B pathway, which should be further investigated in future clinical studies of minocycline against ovarian cancer.

Numerous studies have found that the function of NF-*κ*B not only stimulates the growth of tumor cells, prevents apoptosis, and induces angiogenesis [42, 43], but also contributes to epithelial-mesenchymal transformation (EMT), making metastases faster (through matrix metalloproteinases [MMP] upregulation) [44]. In line with this, minocycline prevents NF-*κ*B activation and nuclear translocation, which inhibits the target-gene expression of MMP-9 in breast and ovarian cancer cells, resulting in reduced cell fusion frequency [33, 35].

Cancer growth and metastasis are dependent on angiogenesis and lymphangiogenesis in a rapid growth process by chemical signals from tumor cells [45]. Angiogenesis is a natural, dynamic, and biomolecular-controlled mechanism initiated throughout the body [46]. Vascular epidermal growth factor (VEGF), as an influential angiogenic factor in neoplastic tissues, has been widely studied in the field of neoplastic vascularization [47]. It has been shown that minocycline could suppress endothelial cell neovasculogenic activity by reducing VEGF secretion from cancer cells [48]. Besides, a combination of minocycline and irinotecan reduces VEGF and IL-8 cytokine induction, thus enhancing the anticancer effects of minocycline alone or in combination with an alkylating agent [49]. Another study has reported that minocycline exerts antiangiogenic effects in the inhibition of human aortic smooth muscle cells (SMCs) migration via attenuating VEGF-induced MMP-9 activity and a downregulated phosphoinositide 3-kinase (PI3K)/protein kinase B (Akt) phosphorylation [50, 51], which suggests that minocycline may be a potential therapeutic agent to suppress angiogenesis, in an MMP-dependent fashion. Conversely, Beadling et al. have shown that minocycline inhibits angiogenesis by a non-MMP-dependent mechanism *in vitro* [52].

Oxygen tension is considered to be another critical factor in the maintenance of angiogenesis in healthy cells, and endothelial cells (ECs) and SMCs are subjected to different oxygen-sensing mechanisms, such as oxygen-sensitive NADPH oxidases, endothelial NO synthase (eNOS), and heme oxygenase [53]. Vascular cells often share a separate category of oxygen sensors that interact with hypoxia-inducible factor (HIF), a heterodimeric transcription factor composed of HIF-1*α* and *β* [54]. HIF-1 activates the transcription of genes that are involved in metabolism, tumor progression, cell survival, metastasis, and angiogenesis in cancer. A study has shown that minocycline exhibits antiangiogenic properties through the suppression of HIF-1*α* protein translation [55]. The p53 deficiency is considered to be linked to the increased HIF-1*α* levels, the most common oncogenic mutations in various cancer cells [56]. HIF-1*α* is often regulated through several oncogenic cellular molecules like the Akt/mammalian target of rapamycin (mTOR), which is found to be upregulated in cancer [57]. The Akt/mTOR activation in response to the growth factors and cytokines results in activation of ribosomal protein S6 kinase (p70S6K) and eukaryotic initiation factor 4E-binding protein-1 (4E-BP1) which in turn lead to enhancement of HIF-1*α* mRNA translation and consequently accumulation of HIF-1*α*, inducing angiogenesis [58,59]. In line with these findings, it has been shown that minocycline attenuates HIF-1*α* expression through an increase in p53 and a decrease in Akt/mTOR/p70S6K/4E-BP1 pathway in ovarian cancer, both *in vitro* and *in vivo*, providing new insight into the discovery of drugs for cancer treatment [60]. The Akt/mTOR and MAPK/ERK pathways both are essential signaling molecules, which regulate tumor proliferation, apoptosis, survival, and invasion/metastasis [61]. Notably, the ERK mechanism, at the end of MAPK signaling, plays an integrating role in signaling events stimulating cell growth and proliferation in many mammalian cell types in incorporating foreign signals from mitogens, such as the epidermal growth factor (EGF) [62]. More importantly, the drug resistance gained from inhibitors of upstream kinases could be overcome by ERK-inhibitors [63]. Mechanistically, minocycline enhances mitomycin C-induced cytotoxicity through downregulating ERK1/2-mediated Rad51 expression in human non-small cell lung cancer cells [64]. Interestingly, the safety and effectiveness of minocycline in patients with EGFR mutated lung cancer, together with tyrosine kinase inhibitors, are ongoing by Thottian et al. [65].

Centered attention has also been focused on cell cycle arrest and apoptosis in cancer therapy [66]. Cancer cells have several primary regulating elements of the cell cycle and apoptosis that affect cyclin-dependent kinases expression, such as the family of Bcl-2 proteins, p53 protein, and the inhibitor of apoptosis proteins (IAPs) [67]. In cellular responses to DNA damage, and cell cycle and apoptosis regulation, p53 plays a vital role among these signaling pathways [68]. It has been well established that p53 acts as a transcription factor in genes that are important to cell cycle control (e.g., p21) or apoptosis (e.g., Bax, Bak, PUMA, and Bcl-2) [69]. In line with these mechanisms, Pourgholami et al. have shown that minocycline prevents the growth and development of ovarian cancer cells, suppresses DNA synthesis, and downregulates cyclins A, B, and E, which results in cells being arrested in the G0 cycle. The exposure of cells to minocycline results in DNA laddering, caspase-3 activation, and PARP-1 cleavage, as well [70].

Notably, minocycline suppresses tumor differentiation and tumor growth *in vivo* [70]. In leukemia cells, Fares et al. and Song et al. have shown that minocycline exerts cytotoxic effects and, consequently, apoptosis through DNA damage, lysosomal degradation, Bcl-xL deamidation, and mitochondria-mediated and caspase-dependent pathways, indicating a therapeutic potential of minocycline in treating leukemia [14, 71]. Ruiz-Moreno et al. in their recent study on acute lymphoblastic leukemia Jurkat cells have shown that minocycline induced apoptosis through the H_2_O_2_-mediated signaling pathway. Also, the apoptosis-inducing effects of minocycline are dependent on the activation of transcription factors and proapoptotic proteins Bax/PUMA. Because of the harmless impacts of minocycline on human peripheral blood lymphocyte cells, it has been suggested that minocycline has promising effects against leukemia [72]. Notably, in hepatocellular carcinoma, a combination of minocycline and cisplatin exerts synergistic effects on growth inhibition through apoptosis induction and S-phase cell cycle arrest [73].

Autophagy has been originated during the last decade as an essential mechanism in controlling homeostasis [74]. Autophagy can also be induced by tissue damage stress or highly developed tumors, in addition to maintaining normal homeostatic processes [75]. Autophagy modulates a cellular reaction to alterations inside and outside cells during tumor growth, leading to adaptation of the tumor [76]. While autophagy regulation seems a well-defined natural mechanism, it remains an essential topic for research in the field of cancer progression [77]. In this regard, it has been shown that minocycline activates cell autophagy through the beclin-1 signaling pathway and increases the antitumor activity of cisplatin (as an alkylating agent) in Hep-2 larynx carcinoma cells [78]. Accumulating evidence has shown that autophagy is a regulator of immune response and inflammation [79]. Minocycline has been shown to exert an immunomodulatory impact on cytokine production [80]. Minocycline has also been documented to induce autophagy, thereby affecting inflammatory and immune responses [81]. In this regard, Sun et al. have shown that minocycline, by inhibiting mTOR, induces autophagy, consequently inhibits cytokine production and cell proliferation, and protects against LPS-induced toxicity in human monocytic leukemia cells (THP-1). Additional research has suggested that crosstalk can be involved in the modulation of inflammatory responses between the inhibitor kappa-B kinase (IKK)/NF-*κ*B signaling pathway and autophagy flux [82]. Furthermore, rapamycin, an mTOR inhibitor, cooperatively acts to reduce TNF-*α* and causes autophagy by blocking mTOR with minocycline [83].

Retinoic acid (RA) has been recently suggested to provide a standard mode of anti-inflammatory action with tetracycline antibiotics. It is a vital and extremely potent endogenous retinoid, having pronounced anti-inflammatory properties and antiacne activity [84]. The growth of many human tumor cells was shown to be inhibited by RA [85]. RAs have been shown to be physiologically controlling regulators of embryonic development, vision, fertility, bone-forming, hematopoiesis, differentiation, and growth [86]. The RA function is primarily regulated by members of the subfamily of RA receptor (*R*) *α*, RAR*β*, and RAR*γ*, which belong to the superfamily of transcription factors in the nuclear receptor [87]. Overexpression of RAR*γ* plays a role in the growth and differentiation of tumor cells through nongenomic activation of the PI3K/Akt and NF-*κ*B signaling pathways [88, 89]. RARs have also been identified as cell growth modulators, differentiation, and apoptosis [90]. Abnormal expression and function of RARs, in particular, are frequently involved in cancer growth, inflammation, and development [91]. It has been shown that the anti-inflammatory effects of minocycline are mediated by RAR signaling [92]. Furthermore, Regan et al. have shown that the growth-inhibitory mechanism of minocycline on human prostate cancer cells was through triggering the RAR signaling pathway [93].

### 2.1. The Role of Minocycline in GBM

Microglia are resident immune cells of the brain that release proinflammatory cytokines when activated. Abundant tumor mass cells are microglia and macrophages [94]. In this regard, the GBM microenvironment has an enormous impact on tumor development and dissemination [95]. Increasing evidence suggests that GBM attracts specific cell populations and indicates that microglia and macrophages are activated, promoting the growth of tumor cells [96]. The presence of two phenotypically distinct cell groups classed as M1 and M2 macrophages is another important determinant of the characteristics and function of glioma-associated microglia (GAM) and macrophages. M1 macrophages are typically activated and produce a response to tumors via immune system activation, reactive oxygen species (ROS), NO, or proinflammatory cytokines, such as TNF [94,97]. Conversely, M2 macrophages perform immunosuppressive functions such as IL-10 releases and tumor progression and cause metastatic processes by facilitating angiogenesis and extracellular degradation of the matrix [98]. GAM also have the M2 phenotype similar to macrophages. Indeed, a change in the M1 to M2 phenotype during tumor progression is associated with the escaping of tumor cells from the immune system [99].

Minocycline has been shown to inhibit the M1 polarization of microglia selectively [100]. Interestingly, minocycline attenuates microglial activation and blocks the long-term epileptogenic effects of early-life seizures [100]. Regarding the anti-inflammatory effects of minocycline, it has been shown that this promising agent reduces prostaglandin E synthase expression and 8-isoprostane formation in LPS-activated primary rat microglia [101]. A similar study has demonstrated that minocycline could prevent cerebral ischemia-induced neuroinflammation by the suppression of microglial activation [102]. Considering that minocycline suppresses inflammation by inhibiting microglial activation and cytokine production in CNS disease models [103,104], the anti-inflammatory effects of minocycline are ongoing in phase II randomized placebo-controlled study [105]. Clemens et al. have shown that the anti-inflammatory effect of minocycline is via inhibition of local RA turnover in human microglial-like cells [92].

As discussed, the GBM invasion is enhanced through the expression of MMPs in the microenvironment, which degrades the ECM [106]. GBM cells release soluble factors to stimulate membrane type 1 metalloproteinase (MT1-MMP) expression in tumor-related microglia and then activate cell invasion and metastasis, through MMP-2/MMP-9/MT1-MMP-induced matrix degradation [107]. Hence, GBM is among the enormous challenges of brain tumor treatments, leading to poor patient survival. In this regard, minocycline, a highly lipophilic microglia inhibitor with an excellent BBB penetration property, has recently been proven to be a promising novel candidate for adjuvant therapy against malignant GBM since it reduced GBM growth both *in vitro* and *in vivo*, by an attenuation in the expression of protumorigenic effects of MT1-MMP as well as a significant inhibition in p38 MAPK expression (responsible for MT1-MMP upregulation in microglia) [108]. Besides, the inhibition of p38 MAPK has also been shown to reduce the secretion of proinflammatory cytokines from microglia and tumor cells, resulting in a decrease of GBM migration [109].What is more, minocycline, as a p38 MAPK inhibitor, appears to counteract the protumor phenotype of microglia and reduce tumor growth *in vitro* and *in vivo* by inhibiting downstream microglial MT1-MMP expression in mouse models. The decrease in MT1-MMP expression is, in turn, associated with decreased MMP-2 activity, mitigating metastasis [39].

The expression of MT1-MMP in GAM is caused by chemokine factors released from GBM cells and it behaves via Toll-like receptor 2 (TLR2) and the p38 MAPK signaling cascades. It has been shown that minocycline interferes with p38 MAPK and inhibits the MT1-MMP expression in microglia. Hu et al. demonstrated that the upregulation of MMP-9 and TLR2 was attenuated by minocycline and a p38 MAPK antagonist *in vitro* [110]. Since TLR2 deficiency attenuates the upregulation of microglial MMP-9, it has been proven that TLR2 plays a vital role in GBM targeted-therapy [111].

Besides, TLR2 promotes the development and progression of human GBM via increasing autophagy [111]. In line with this, the autophagy-inducing mechanism of minocycline [112] could be explained by targeting the TLR2 signaling pathway [112]. Accordingly, Liu et al. have shown that the suppression of cell growth by minocycline is mediated by acidic vesicular organelles development in the cytoplasm, autophagic cell death, and endoplasmic reticulum stress-induced apoptosis in GBM cells [112,113] However, based on Desjarlais et al.'s study, it has been found that part of the minocycline's effects on autophagy could be exerted through the inhibition of MT1-MMP [114]. Besides, inhibiting tumor growth in C6 glioma cell xenograft tumor models was reported by minocycline. This was related to autophagic cell death induction, although minocycline still triggers cell death by caspase-3 activation when autophagy is inhibited [112]. In line with the apoptosis mechanisms of minocycline, it has been shown that minocycline prevents primary neurons from radiation-induced apoptosis and promotes radiation-induced autophagy *in vitro* [115]. These experimental data further suggest that minocycline has improved its antimetastatic and antitumor effectiveness through antiproliferative, autophagy-inducing, antiangiogenic, MMP inhibitory, and apoptosis-inducing functions.

Based on a literature survey, minocycline also is expected to prevent the phosphorylation of STAT-3, a transcription factor downstream of EGFR. Minocycline effectively inhibits activation of EGFR-driven STAT-3 in U373 GBM cells at a concentration of 100 *μ*M [116]. The findings of Bow et al. have shown that local minocycline (minocycline polymers), as an angiogenesis-inhibitor, could maximize the median survival (an improvement of 38% in median survival) in the radiation therapy plus oral TMZ combination treatment on rat 9L GBM model [117]. Glial acid fibrillary protein (GFAP), as an intermediate filament, is expressed in mature astrocytes in the CNS. It has been shown that GFAP is upregulated in astrocytoma and GBM, indicating its potential in the prognosis of patients. Recently, Cai et al. have demonstrated that minocycline reduces astrocytic reactivation and neuroinflammation through GFAP, COX-2, NF-*κ*B, IL-1*β*, and TNF-*α* downregulation [118].

**Figure 1:**
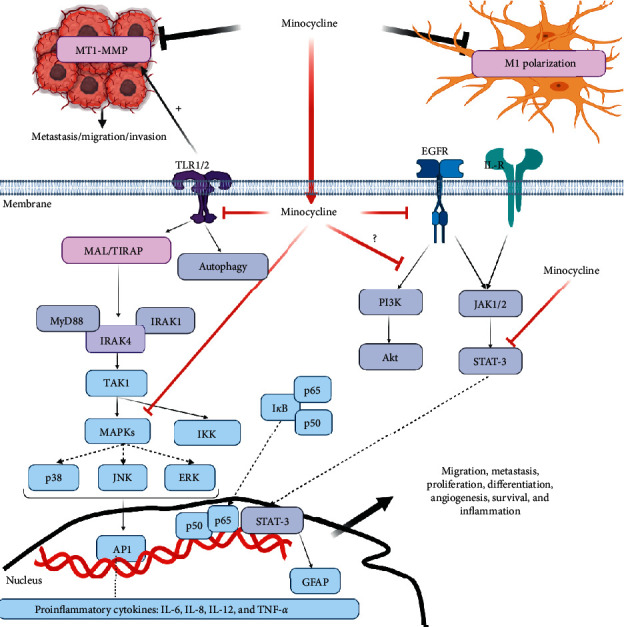
The proposed mechanisms of action of minocycline against GBM.

As mentioned, minocycline could inhibit the PI3K/Akt signaling pathway in various tumor cells [50,51]. In contrast, a study has demonstrated that minocycline activation of PI3K p110*α* results in cell dysfunction and cell mortality in 9L glioma cells. The findings have proven that PI3K*α* activation is essential for minocycline-induced GBM cell death [119]. This result raises serious questions here concerning the development of PI3K inhibitors in treating cancer, especially GBM. Collectively, as revealed in Figure 1, we have summarized the molecular targets of minocycline in GBM.

## 3. Conclusions

During the last 30 years, GBM treatment of clinical trials has now become multimodal, highlighting the improvement in patient survival through adjuvant chemotherapy following maximal surgery [120]. This progress with therapeutic procedures has, unfortunately, had a minimal impact on patient survival [121]. It is, therefore, urgently necessary to explore a new strategy to combat GBM.

Minocycline, as a semisynthetic tetracycline, has been used for several years as an antibiotic for acne vulgaris, perioral disease, and dermatological sarcoidosis. Currently, minocycline is used to control inflammatory conditions because of its immunomodulatory, anti-inflammatory, and neuroprotective activities. Several animal models studies and several clinical trials have confirmed minocycline to be a promising therapeutically chemical agent for cancer, especially GBM, alone or combined with other medications (Table 1).

Minocycline has been proposed to have several anticancer mechanisms of action in GBM, including inhibition of proinflammatory and metastatic enzymes, such as iNOS and MT1-MMP signaling functions, GFAP, COX-2, NF-*κ*B, IL-1*β*, and TNF-*α* downregulation, autophagic cell death induction, caspase-3 activation, suppression of microglia activation, inhibition of M1 microglia polarization, p38 MAPK inhibition, inhibition of EGFR-driven STAT-3 activation, and targeting the TLR2 signaling pathway. Minocycline also possesses some promising effects that are worthy of attention in GBM therapy: (1) it is a well-absorbed, safe, and a long-acting drug in humans with low adverse effects, (2) it is relatively inexpensive, and (3) it crosses BBB owing to its small size and the highest lipophilicity among tetracyclines. The established role of minocycline as a conventional antibiotic throughout years indicates that it could be a valuable option in GBM therapy through exerting its promising antitumor properties. However, a greater understanding of the mechanisms underlying the antitumor effects of minocycline as well as supportive evidence from in vivo studies and clinical trials is needed to assess the therapeutic potential of this antibiotic in a more accurate fashion. The current positive findings suggest that minocycline could be a potential cost-effective drug to combat many of the malignant disorders.

## Figures and Tables

**Table 1:** The effects of combination therapies-based minocycline against GBM.

Combination therapy	Type of study	Pharmacological effect (s)	Reference
Minocycline + MJ-66^1^	Intracranial GBM xenograft	(1) Inhibition of cell growth	[122]
		(2) Induction of cell death	
		(3) Aggravation of DNA damage	
Minocycline + bevacizumab	Clinical trial^2^	Ongoing	[123]
Minocycline + TMZ	Clinical trial^3^	Ongoing	[124]
Minocycline + STAT3 inhibitor	In vitro and in vivo	(1) Reducing cell viability	[125]
		(2) Suppression of tumor growth	
Minocycline^4^ + BCNU^5^	Rodent brain tumor model^6^	(1) Inhibition of tumor growth	[126]
		(2) Impairing microglia activation	
Minocycline + telmisartan + zoledronic acid	In vitro	(1) Inhibition of MCP-1 expression	[127]
		(2) Interfering with glioblastoma growth	
Minocycline + sulforaphane	In vitro^7^	Inhibition of microglial activation	[128]
CUSP9v3^8^	Clinical trial^9^	Ongoing	—

^1^A synthetic quinazolinone analog. ^2^A phase 1 study for recurrent GBM patients (NCT01580969). ^3^A phase 1 study for newly diagnosed GBM patients (NCT02272270). ^4^A biodegradable controlled-release polymer. ^5^Systemic injection. ^6^Local delivery of minocycline (intracranially). ^7^Microglial cells. ^8^Nine repurposed drugs (aprepitant, auranofin, captopril, celecoxib, disulfiram, itraconazole, minocycline, ritonavir, and sertraline) combined with metronomic TMZ. ^9^A phase 1/2 study for recurrent GBM patients (NCT02770378). GBM, glioblastoma multiforme; TMZ, temozolomide; STAT-3, signal transducer and activator of transcription-3; BCNU, carmustine; MCP-1, monocyte chemoattractant protein-1.

## Abbreviations

MAPK: Mitogen-activated protein kinase
PI3K: Phosphoinositide 3-kinase
TLR2: Toll-like receptor 2
EGFR: Epidermal growth factor receptor
MT1-MMP: Membrane type 1 metalloprotease
GFAP: Glial fibrillary acidic protein
I*κ*B: Inhibitor of kappa-B
IL: Interleukin
MAL: MyD88 adaptor like
MyD88: Myeloid differentiation primary response 88
TAK1: Transforming growth factor *β*-activated kinase 1
IRAK: Interleukin receptor-associated kinase
ERK: Extracellular signal-regulated kinase
JAK: Janus kinase
JNK: c-Jun N-terminal kinase
p38: Protein 38
AP-1: Activated protein-1
STAT-3: Signal transducer and activator of transcription 3
TNF-*α*: Tumor necrosis factor *α.*
